# Computational study of diffraction image formation from XFEL irradiated single ribosome molecule

**DOI:** 10.1038/s41598-024-61314-w

**Published:** 2024-05-09

**Authors:** Michal Stransky, Juncheng E, Zoltan Jurek, Robin Santra, Richard Bean, Beata Ziaja, Adrian P. Mancuso

**Affiliations:** 1 European XFEL, Holzkoppel 4, 22869 Schenefeld, Germany; 2 Institute of Nuclear Physics, Polish Academy of Sciences, Radzikowskiego 152, 31-342 Krakow, Poland; 3 Institute of Physics, Czech Academy of Sciences, Na Slovance 2, 182 21 Prague 8, Czech Republic; 4 Center for Free-Electron Laser Science CFEL, Deutsches Elektronen-Synchrotron DESY, Notkestr. 85, 22607 Hamburg, Germany; 5 The Hamburg Centre for Ultrafast Imaging, Luruper Chaussee 149, 22761 Hamburg, Germany; 6 Department of Physics, Universität Hamburg, Notkestr. 9-11, 22607 Hamburg, Germany; 7 Diamond Light Source, Harwell Science and Innovation Campus, Didcot, Oxfordshire, OX11 0DE UK; 8 Department of Chemistry and Physics, La Trobe Institute for Molecular Science, La Trobe University, Melbourne, VIC 3086 Australia

**Keywords:** Atomic and molecular interactions with photons, X-rays, Imaging techniques, Macromolecules and clusters

## Abstract

Single particle imaging at atomic resolution is perhaps one of the most desired goals for ultrafast X-ray science with X-ray free-electron lasers. Such a capability would create great opportunity within the biological sciences, as high-resolution structural information of biosamples that may not crystallize is essential for many research areas therein. In this paper, we report on a comprehensive computational study of diffraction image formation during single particle imaging of a macromolecule, containing over one hundred thousand non-hydrogen atoms. For this study, we use a dedicated simulation framework, SIMEX, available at the European XFEL facility. Our results demonstrate the full feasibility of computational single-particle imaging studies for biological samples of realistic size. This finding is important as it shows that the SIMEX platform can be used for simulations to inform relevant single-particle-imaging experiments and help to establish optimal parameters for these experiments. This will enable more focused and more efficient single-particle-imaging experiments at XFEL facilities, making the best use of the resource-intensive XFEL operation.

## Introduction

Single particle imaging (SPI) at atomic resolution is one of the most vaunted research goals for X-ray free-electron laser (XFEL) facilities^1–3^. Such a capability has the potential for a transformative impact in biological and medical sciences, as high-resolution structural information on biosamples is essential for many research areas therein. This is because the structure of many biologically important particles (macromolecules, viruses, etc.) has not yet been sufficiently explored with conventional X-ray diffraction techniques, due to the lack of successful crystallization^4^. Nevertheless, key challenges to this method remain, and computational explorations of its viability and best use are helpful to optimize the technique towards being valuable to samples of scientific interest^4^.

High-resolution single-particle cryo-EM has been largely developed to reach atomic resolution with new detector technology and image processing algorithms in the past few years^5,6^. However, its limitations in the sample environment and temporal resolution prevent it from investigating the dynamics of proteins under physiological conditions, where the XFEL has the potential^7^.

While the serial femtosecond crystallography (SFX) method^8–12^, which operates on nano- and micro-crystals, and therefore bridges between conventional crystallography and SPI, has proven to be very successful, high-resolution imaging of single non-periodic objects still remains a challenge^4^. Therefore, theoretical studies are necessary to guide further development of single-particle imaging techniques towards their optimization, and also for identifying and overcoming the existing physical and technical limitations of SPI (see, e.g. Ref.^13^).

Such an effort has already led to the development of the start-to-end (S2E) computer simulation pipeline dedicated for computational studies of SPI at the European XFEL facility^14,15^. The pipeline was later developed into the SIMEX platform^16^ for multidisciplinary applications, and a simplified python interface SimEx-lite^17^ for easy user access was added. In Ref.^18,19^, this platform was used to investigate the SPI of a small hydrated protein molecule, 2NIP, containing around 5000 non-hydrogen atoms. However, a realistic SPI study of biological objects containing at least hundreds of thousands of atoms (i.e. of scientific relevance and size which can yield sufficiently strong signal during experimental imaging studies), could not be performed so far with this simulation tool, due to the too-high computational costs. As straightforward extrapolation of SIMEX predictions for small samples to large samples is not possible, due to the differently progressing radiation damage in small and large samples (e.g., Ref.^20^), dedicated computer simulations are necessary to explore this regime. In this paper, we perform a comprehensive analysis of radiation damage during a realistic SPI study of a ribosome macromolecule, containing 142,429 non-hydrogen atoms. For the simulation, we use the SIMEX framework^16^, with a dedicated software for modeling X-ray induced radiation damage in large finite-size samples, the tree-code-extended XMDYN tool^21–23^. With these codes, we estimate the minimal number of simulated molecular-dynamics realizations of the X-ray irradiated molecule (performed under typical experimental conditions) needed to reliably compute its time-integrated and realization-averaged diffraction image. We compare the actual prediction to that obtained previously for the small 2NIP protein in Ref.^18^. We do the same for the prediction on the measure of degradation of diffraction image quality: the R-factor. Discussion and outlook then follow.

Our results demonstrate the feasibility of computational SPI studies for biological samples of realistic size. This is very important as it shows that the SIMEX platform can be used for simulations of SPI experiments preceding practical experiments, and help to establish optimal parameters for these experiments. This can stimulate more efficient SPI studies at XFEL facilities worldwide.

## Simulation setup

### Simulation framework

**Figure 1:**
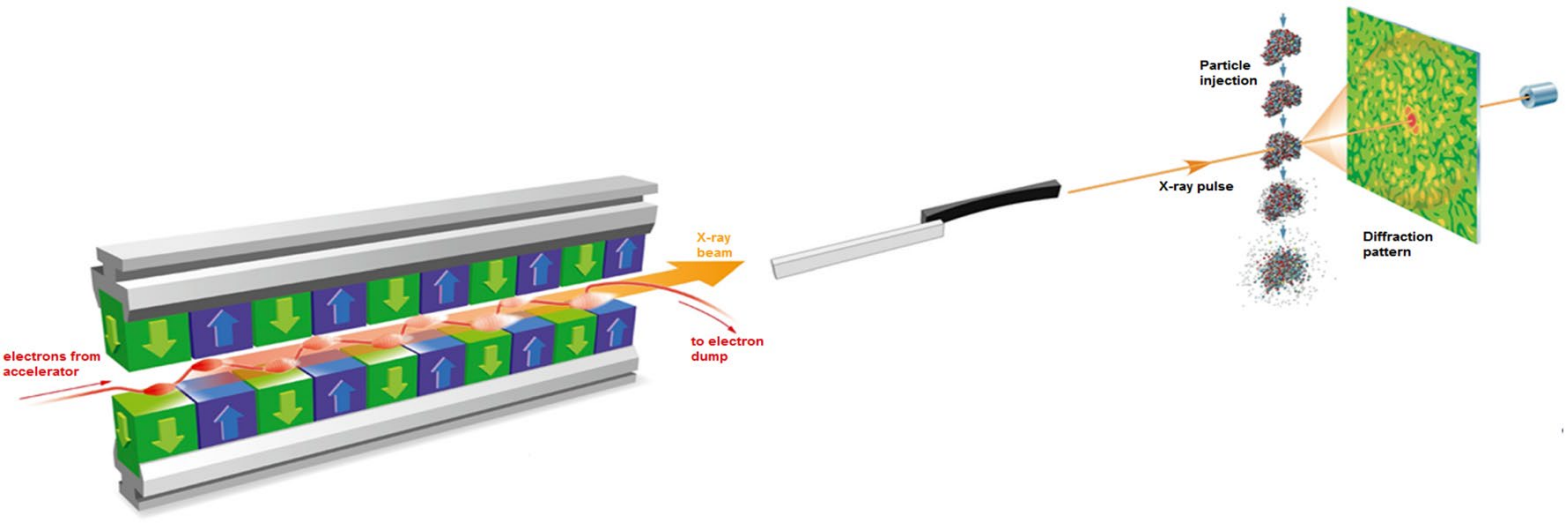
Schematic of a typical single-particle imaging experiment, modeled within our start-to-end simulation framework^18^. X-rays propagate from the source to the sample through the beamline optics and then interact with the sample. The scattering pattern is recorded by the detector on the right.

The S2E/SIMEX modeling framework was discussed in detail in Ref.^16,18^. In short, an SPI experiment (Fig.1) is modeled using a virtual simulation pipeline. It consists of consecutive modules providing: (1) simulations of SASE X-ray pulses (the X-ray source), (2) description of beam propagation through the XFEL optics, (3) modeling of the interaction between X-rays and the irradiated sample, (4) formation of X-ray scattering patterns, (5) processing of individual diffraction patterns, and (6) real-space structure determination from X-ray scattering patterns assembled in the reciprocal space.

### Irradiation conditions

For our simulations, we used the set of 55 SASE X-ray pulses generated for our earlier studies^15,18^. The nominal X-ray pulse parameters were: 4.96 keV photon energy, $5 \times 10^{11}$ photons per pulse, 9 fs FWHM pulse duration and 250 nm $\times$ 160 nm FWHM focal size. The simulation parameters match the ones from the earlier simulation work^15^ to aid comparison. The values represent an achievable, though not optimal performance of the accelerator and SPB/SFX instrument at the European XFEL.

### Molecule description

Ribosomes are key molecules in living cells. They are responsible for assembling amino acids into protein chains, using information carried by m-RNA molecules. Ribosomes consist of a large subunit and a small subunit. In some studies the subunits are investigated separately; in this study, however, the entire assembly is considered. The imaged ribosome is taken from the E. Coli bacteria, indexed as 4V6C in the PDB database^24^. It includes 243,324 atoms in total, out of which 100,895 atoms are hydrogen atoms. The largest atom-atom separation is about 300 Å which defines an upper limit for the size of this inhomogeneous molecule of irregular shape.

### X-ray—molecule interaction

We used the tree-code-extended XMDYN code^21–23^ to simulate the dynamics of the irradiated ribosome molecule. XMDYN follows the ionization dynamics of atoms and ions, using a Monte Carlo scheme combined with first-principle atomic-structure calculations. When an orbital of an atom is ionized, the corresponding occupation number is updated and all orbitals of the atom are reoptimized. Simultaneously, an electron is ejected in the immediate vicinity of the ionized atom. The ejected electron is then treated as a classical particle.

XMDYN captures the real-space dynamics of the atoms/ions and of free electrons using the molecular dynamics (MD) scheme. Only Coulomb forces between charged particles are considered because chemical bonds are expected to break up early in the exposure due to the rapid sample ionization. As atoms and electrons are treated as classical particles and information on the specific atomic configuration of each ion is provided by the code, one can easily calculate scattering patterns from the atomic snapshots.

The ribosome molecule containing over two hundred thousand atoms and a very high number of excited electrons could not be simulated using the original *n*-body solvers for Coulomb interaction and secondary ionization implemented in XMDYN. Both of the solvers had O($n^2$) computation time complexity (where *n* is the number of particles). In order to simulate such a large system, we incorporated the Pretty Efficient Parallel Coulomb-solver (PEPC) developed in Forschungszentrum Jülich^25^, and developed a more efficient secondary ionization solver (based on tree code search for nearest neighbors), reducing the time complexity to O($n \cdot \textrm{log}(n)$). A detailed description of the solver can be found in Ref. ^23^.

In total, we have generated 100 different molecular-dynamics realizations (trajectories) of the stochastic dynamics within the X-ray irradiated ribosome molecule, randomly oriented with respect to the incoming X-ray beam. Calculation of one MD trajectory took about 45 days. Calculation of all MD trajectories took about 17,000 CPU days.

## X-ray induced radiation damage in ribosome

During the exposure to the 9 fs FWHM duration free-electron laser X-ray pulse, atoms undergo photoionization from core levels, with subsequent Auger decay of core holes. The released electrons cause further radiation damage by collisional ionization of atoms/ions. Incoming X-rays scatter coherently and incoherently on the electrons bound on atoms/ions within the molecule and incoherently on the free electrons, producing a diffraction image with a fluctuating background which encode the information on the molecule structure.

The ionization process reduces the number of bound electrons. As a result, the coherent scattering signal from the sample decreases (e.g. Ref.^11,26,27^). In addition, ionized atoms start to repel each other due to mutual repulsive Coulomb forces. This leads to atomic displacement and, eventually, to sample expansion. The progressing atomic displacements also reduce the quality of the diffraction image.

**Figure 2:**
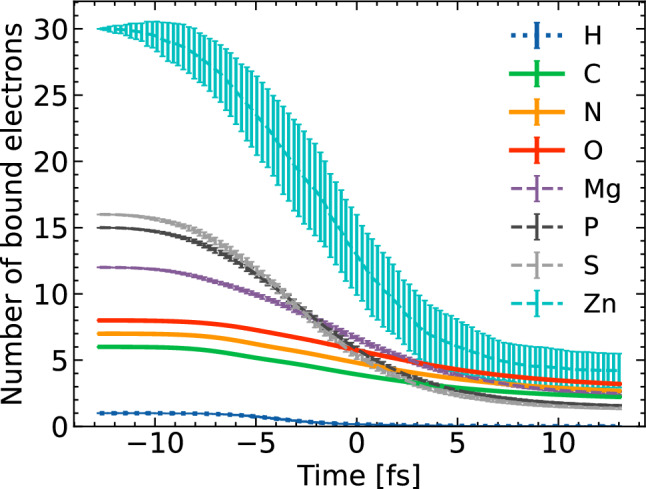
Average number of bound electrons per atom as a function of time calculated for various atomic species within the ribosome molecule. The average was taken from 100 XMDYN simulations. The average over the SASE pulses employed is peaked at 0 fs.

In order to quantify the effect of X-ray induced radiation damage in the ribosome molecule, we first show the average number of bound electrons for each atomic species as a function of time (Fig. 2). The average was calculated from 100 XMDYN simulations, as discussed in the subsection on “X-ray - molecule interaction”.

The most abundant non-hydrogen atoms, C, N and O, yield the strongest contribution to the elastic scattering signal. With increasing time, their ionization progresses very similarly, reducing the scattering cross sections at the time of the pulse maximum to about two thirds of their respective initial values. For time references, we define the time zero here as the time of the maximum of the average temporal envelope of the 55 SASE pulses, each 9-fs long that we used in the simulations. The element zinc is a trace element with only one atom present in the entire molecule. Therefore, it has the largest error bars. However, zinc does not have a measurable effect on the overall scattering signal.

**Figure 3:**
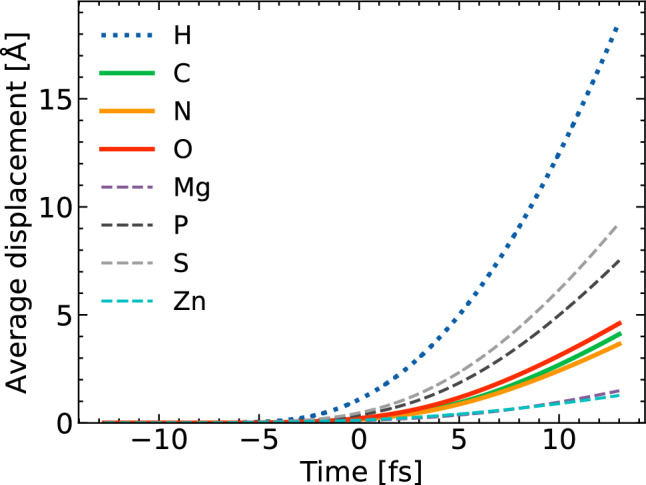
Average atomic displacement calculated for different atomic species within the ribosome molecule. Pulse and simulation parameters are the same as in Fig. 2.

Figure 3 shows average atomic displacements for various atomic species during the X-ray pulse. At the maximum of the pulse (time zero), the sample’s ability to scatter X-rays has already been somewhat reduced due to ionization, so that the strongest contribution to the time-integrated signal registered at the detector occurs shortly before this maximum, yet the displacement for the C, N and O elements up to the time zero is still below 1 Å.

**Figure 4:**
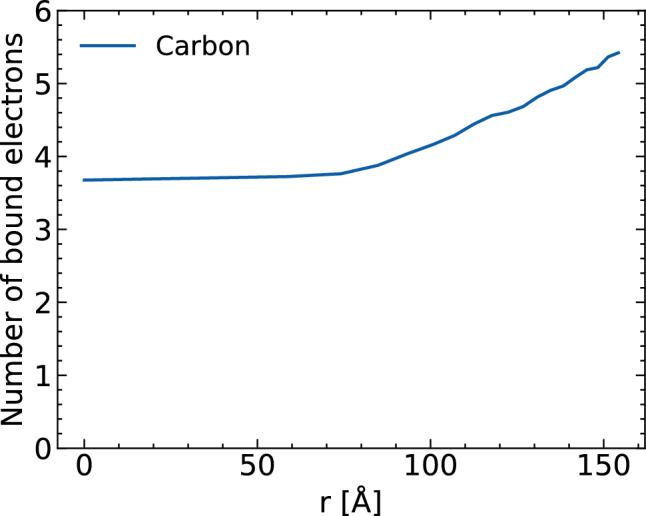
Average number of electrons bound to carbon ions at time zero as a function of distance from the molecule’s center of mass at time zero. Pulse and simulation parameters are the same as in Fig. 2.

In Ref.^18^, the small 2NIP protein was studied. It was observed that the sample ionization was mostly driven by collisional ionization induced by photo- and Auger electrons. The ionization was reduced at the sample edge, due to the electron density gradient. Similar behavior has also been found for the ribosome molecule. Figure 4 shows that at time zero the average number of electrons bound to carbon ions is larger at the sample edge than in its central region. The example of carbon is shown as it is the most abundant non-hydrogen element in biological molecules. Similar behavior was observed for nitrogen and oxygen atoms (not shown here). There are two main reasons for higher ionization degree in the center of molecule: first, electrons ejected close to the edge are more likely to leave the molecule without triggering secondary ionization events and second, slower excited electrons get trapped in the molecule’s central region causing further ionization therein. The resulting non-uniformity in the spatial distribution of bound electrons reduces the quality of imaging in the 50–100 Å resolution region. As mentioned above, this reduced ionization damage at the sample edge is very similar to that of the non-hydrated 2NIP sample discussed in^18^.

**Figure 5:**
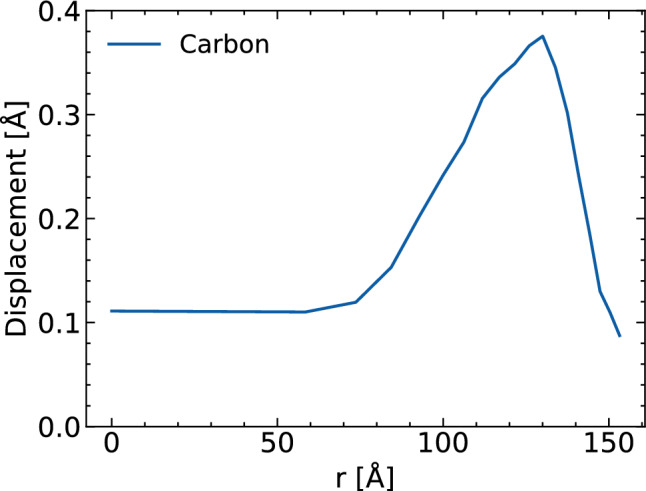
Average displacement of carbon ions at time zero as a function of distance from the molecule’s center of mass at time zero. Pulse and simulation parameters are the same as in Fig 2.

Similar analysis can be performed for the average atomic displacement. Figure  5 shows the average atomic displacement at time zero obtained from 100 XMDYN realizations for carbon atoms/ions.

The displacement is relatively small in the central molecule region, where ion charges are screened by free electrons. In the region close to the molecule edge, it becomes up to three times larger due to the decreased electron density which stimulates surface expansion. However, even then, the displacement still remains only a fraction of an Ångstrom.

## Conditions for statistically reliable description of radiation damage in SPI simulations

During the SPI experiments, one obtains hundreds of thousands of diffraction images. Each image results from X-ray diffraction from a randomly oriented particle, with stochastically progressing radiation damage. It is computationally not possible to simulate that many MD realizations, therefore a reasonable simplification must be applied, in order to reduce the computational effort.

The most natural way is to try to reduce the number of calculated MD realizations. However, in such a case, multiple images must be calculated at different (random) sample orientation, using the same molecular-dynamics realization. This strategy was used in earlier studies^14,18^. However, for ribosome, the XMDYN simulations take a very long time. Therefore, it becomes critical to find a minimal number of MD realizations that yields statistically reliable average diffraction patterns.

In order to establish a lower limit for the number of required MD realizations, we studied the convergence of the average 3D reciprocal-space time-integrated image of the simulated molecule (later referred to as “time-integrated 3D image”, or “3D image”). The analysis performed in the reciprocal space gave us the immediate advantage of addressing directly different resolution regimes.

First, we obtained a time-integrated 3D image for each of the simulated MD realizations. The time integration used the time-resolved photon count rate for the SASE pulse that was used for the calculation of that realization. The time integration was performed using 10 diffraction images taken at 10 selected time steps in the same way as stated in Ref.^18^. They were all calculated on a preselected 3D **q**-grid with the oversampling ratio of 2.8 and the edge full-period resolution of 3.5 Å. At the end, each of the 3D time-integrated images was normalized by dividing the signal by the total incident photon count of the respective pulse. From the 100 real-space realizations obtained for the X-ray irradiated ribosome molecule, we calculated 100 time-integrated 3D-reciprocal-space images. From them, we calculated a mean 3D reciprocal space image on the selected **q**-grid. This 3D image served as the reference image in our analysis.

**Figure 6:**
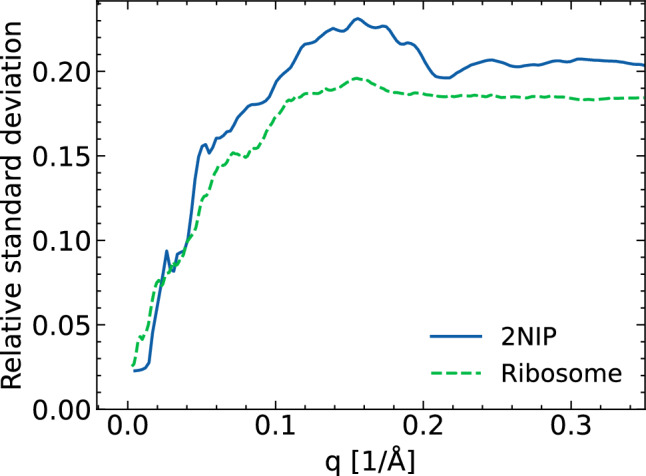
The relative standard deviation of the intensity signal as a function of *q* for 2NIP and ribosome molecules. It was calculated from 100 XMDYN realizations for both molecules.

Further, for each of the voxels on this grid we also calculated the standard deviation of the signal. The voxel intensity varies in the volume over six orders of magnitude; however, the same is true for the estimate of the standard deviation of the voxel intensity, so one is naturally led to define a relative standard deviation in each voxel as $\sigma _{px} / I_{px}$. To facilitate the interpretation of the results, we averaged these relative standard deviations of the signal on spheres of constant *q* and plotted those as a function of *q* (see Fig. 6). This enabled us to better judge the quality of convergence at different reciprocal resolution scales.

In our previous work^18^, we simulated 1000 XMDYN realizations for 2NIP (which has diameter of 70 Å) using the same set of X-ray pulse parameters (same SASE pulses). We took 100 of these realizations, so we could compare the relative standard deviation for molecules of different sizes. In Fig. 6, we show the relative standard deviation of the signal as a function of *q*, obtained for both molecules. The relative standard deviation rapidly increases with increasing *q* and then stabilizes at a value close to 0.2. This implies that if we calculate the mean reciprocal-space image from $N\ge 25$ realizations, the relative standard deviation of this mean would be less than 4% in the entire *q*-range (assuming the validity of the central limit value theorem in this case).

Perhaps surprisingly, the relative standard deviations of the signal are comparable for both molecules, with the relative standard deviations of the signal for 2NIP being slightly higher than that for the ribosome. This implies that one would need a similar number of realizations for both the small 2NIP and the large ribosome, in order to achieve a similar accuracy for the time-integrated 3D diffraction image.

**Figure 7:**
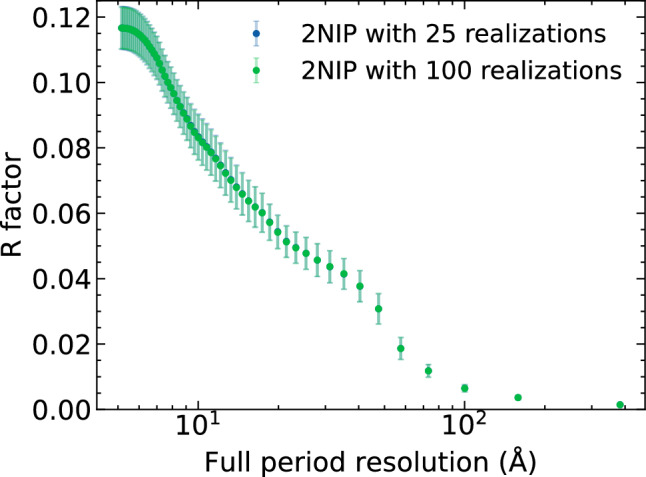
R-factor as a function of full-period resolution. It measures the degradation of diffraction image quality with respect to the diffraction image from undamaged 2NIP molecule. It was calculated with: (i) all 100 simulated MD realizations, and (ii) with 25 realizations randomly selected.

**Figure 8:**
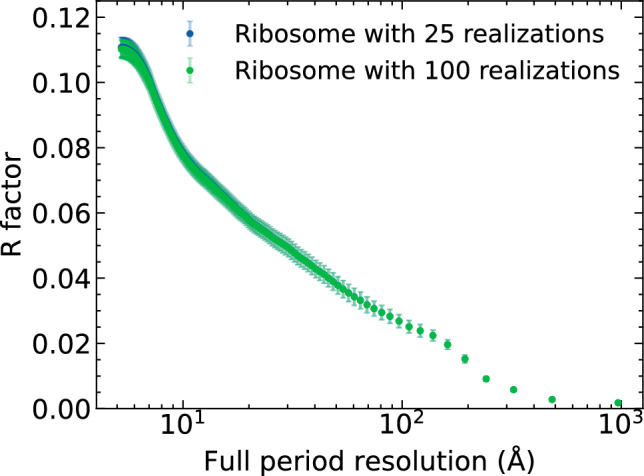
R-factor as a function of full-period resolution. It measures the degradation of diffraction image quality with respect to the diffraction image from undamaged ribosome molecule. It was calculated with: (i) all 100 simulated MD realizations, and (ii) with 25 realizations randomly selected.

Below we also show the resolution-dependent R-factor for ribosome and 2NIP molecules (see Figs. 7, 8 respectively). The R-factor is calculated from reciprocal-space intensity distributions. It measures the degradation of the diffraction image quality due to radiation damage and Compton scattering with respect to the diffraction from an undamaged molecule (elastic signal only; see e.g. Ref.^18^). We found that the R-factor was already converged when 25 MD realizations were used for its calculation, both for the ribosome and 2NIP molecules.

One has to mention that for calculating the R-factor we used 2000 diffraction images. They were created from 100 MD realizations of X-ray irradiated ribosome, after rotating them randomly 20 times for each realization. This was possible due to the observation that the damage of the molecule negligibly depends on the orientation of the molecule with respect to the incoming X-ray beam. This is discussed in detail in the supplementary material. This procedure significantly reduced otherwise very extensive computational costs.

In the previous work^18^, the image quality degradation (due to radiation damage and compton scattering) quantified using the R-factor, was studied for the 2NIP molecule, which contains only $\sim$ 5000 non-hydrogen atoms. Our current analysis has provided the R-factor for a much larger molecule, ribosome, with $\sim$ 150,000 non-hydrogen atoms. As the R-factor values are very similar for both molecules (Figs. 7 and 8) despite the very different molecule size, one can expect that the R-factor for molecules of a size located between these two values will be similar for the same set of X-ray pulse parameters.

## Conclusions

In this work, we studied computationally the conditions for statistically reliable description of radiation damage in SPI simulations, in particular the convergence with respect to the realization number and the image quality degradation. We showed that in order to meaningfully characterize image quality degradation during SPI of small and large molecules, one only needs to simulate a few tens of realizations. Also, we demonstrated that for the same set of X-ray pulse parameters, the R-factors, measuring the image quality degradation, are very similar, both for the $\sim$ 150-thousand-non-hydrogen-atom large ribosome molecule and $\sim$5-thousand-atom large 2NIP molecule. One can then expect that the realization-number convergence and R-factor for a molecule of a size located between these two limiting cases will behave similarly for the same set of X-ray pulse parameters. Our study shows that computational SPI studies for biological samples of realistic size are feasible, and the SIMEX platform can be efficiently used for the simulations of SPI experiments preceding real experiments, helping to estimate optimal parameters for these experiments.

### Supplementary Information

Supplementary Information.

## Data Availability

Data are available from the corresponding author M. S. upon reasonable request.
