# Supplementary material for: Computational study of diffraction image formation from XFEL irradiated single ribosome molecule
Source: Sci Rep. 2024 May 9;14:10617. doi: 10.1038/s41598-024-61314-w (PMC11078940; doi:10.1038/s41598-024-61314-w)
Supplement: Supplementary file 1 — Supplementary Information. [file 41598_2024_61314_MOESM1_ESM.pdf]

Supplementary material: Computational study of diffraction image formation from XFEL irradiated single ribosome molecule

Michal Stransky[1,2,3*], Juncheng E[1*], Zoltan Jurek[4,5], Robin Santra[4,5,6], Richard Bean[1], Beata Ziaja[4,2], and Adrian P. Mancuso[1,7,8*]

[1]European XFEL, Holzkoppel 4, 22869 Schenefeld, Germany
[2]Institute of Nuclear Physics, Polish Academy of Sciences, Radzikowskiego 152, 31-342, Krakow, Poland
[3]Institute of Physics, Czech Academy of Sciences, Na Slovance 2, 182 21 Prague 8, Czech Republic
[4]Center for Free-Electron Laser Science CFEL, Deutsches Elektronen-Synchrotron DESY, Notkestr. 85, 22607 Hamburg, Germany
[5]The Hamburg Centre for Ultrafast Imaging, Luruper Chaussee 149, 22761 Hamburg, Germany
[6]Department of Physics, Universität Hamburg, Notkestr. 9-11, 22607 Hamburg, Germany
[7]Diamond Light Source, Harwell Science and Innovation Campus, Didcot, Oxfordshire OX11 0DE, UK
[8]Department of Chemistry and Physics, La Trobe Institute for Molecular Science, La Trobe University, Melbourne, VIC 3086, Australia
[*]Correspondence and requests for materials should be addressed to: michal.stransky@xfel.eu, juncheng.e@xfel.eu, adrian.mancuso@diamond.ac.uk

Dependence of radiation damage in ribosome molecule on the molecular orientation with respect to the X-ray beam direction

Here we analyze how strongly the radiation damage is affected by the ribosome molecule's orientation with respect to the direction of the incoming X-ray beam. Below we show the distribution of the difference between the actual number of bound electrons in an atom and its average value obtained for all non-hydrogen atoms in the ribosome molecule at time zero (i.e., the maximum of the X-ray pulse) (Fig. S1). The analyzed ensemble consisted of 10 randomly selected MD realizations. We compare two cases: (i) 10 realizations with fixed initial orientation of the molecule, and (ii) 10 realizations with random orientation. The resulting curves are almost overlapping, i.e., practically no effect of molecule orientation is observed. Note that due to a large number of atoms in the ribosome molecule, the set of 10 MD realizations already gives reliable statistics.

Similar comparison can be done for atomic displacements as they also influence the quality of the diffraction image. The actual displacement of a chosen atom is compared to its average displacement, calculated from 10 MD realizations in two cases: (i) with fixed molecule orientation, and with (ii) random molecule orientation. The distribution profiles in Fig. S2 are almost identical, indicating no effect of the sample orientation of the displacement distribution.

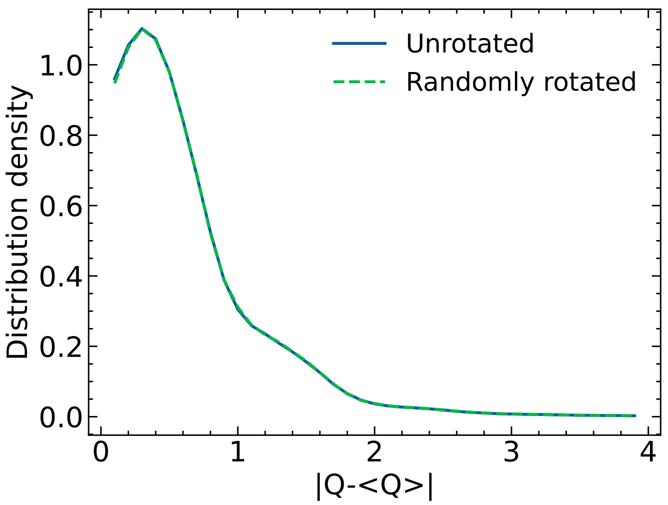

Figure S1. Distribution of absolute differences between the number of bound electrons in a a chosen atom and its average value, derived for 10 MD realizations of unrotated molecule and 10 MD realizations with randomly oriented molecule, and averaged over all atoms contained in the ribosome molecule.

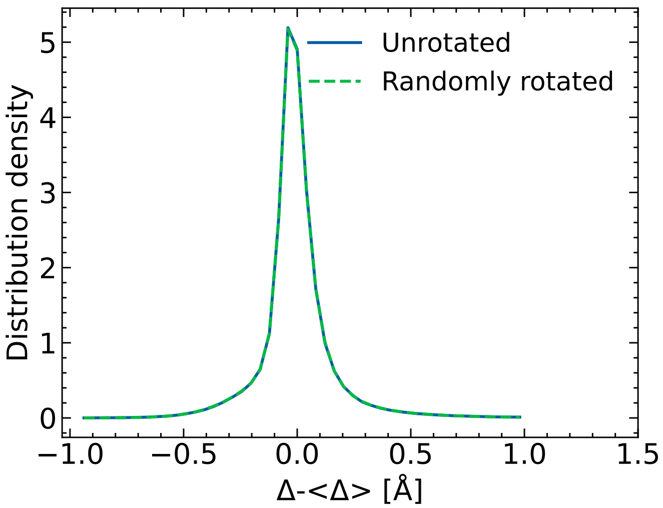

Figure S2. Distribution of differences between displacement of a chosen atom and its average value, derived for 10 MD realizations with unrotated molecule and 10 MD realizations with randomly oriented molecule, and averaged over all atoms contained in the ribosome molecule.
